# Expressing the Differences between Crohn Disease and Ulcerative Colitis

**DOI:** 10.1371/journal.pmed.0020230

**Published:** 2005-08-30

**Authors:** Cisca Wijmenga

## Abstract

Wijmenga discusses the implications of a study that used a comprehensive gene expression approach to find new genes and pathways relevant to the pathophysiology of inflammatory bowel disease.

Inflammatory bowel disease (IBD) is mainly seen in developed, urbanized countries, and its incidence increased steeply at the beginning of the 20th century, concurrently with improved hygiene. Similar trends have been observed for allergic and autoimmune disorders, suggesting that a reduction in microbial burden contributes to disease pathogenesis. Apart from environmental factors playing a strong role, genetic factors are also involved in IBD, so it is regarded as a complex disorder.

IBD can be classified as Crohn disease (CD) or ulcerative colitis (UC). Although these two forms of IBD share similar clinical and pathological features, the disease is heterogeneous, with marked differences in clinical presentation, underlying genetic factors, and response to treatment. The differentiation between CD and UC has been debated for a long time. Elucidation of the underlying disease pathway(s) may help to resolve this debate and also provide important leads for novel therapeutic targets. Recent genetic studies have been fairly successful in identifying disease genes for CD, in particular, pointing toward an impaired integrity of the epithelial barrier (reviewed in [1]). However, a broad view of the underlying pathogenic mechanism in IBD is expected to come from complementary approaches, including gene expression profiling using microarray studies.

## Molecular Profiling to Identify the Molecular Mechanisms Underlying IBD

**Figure 1:**
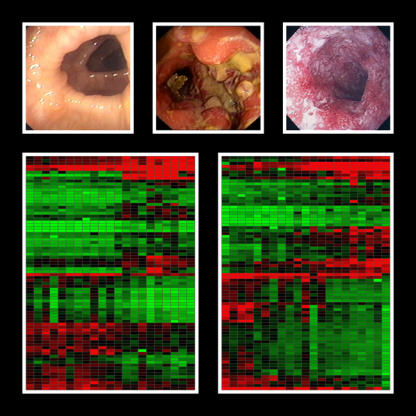
Colonoscopy Images Above are colonoscopy images from a healthy control patient with a noninflamed colon (left) and from patients with highly inflamed CD (middle) and UC (right). Below are resulting heat maps of differentially expressed genes identified for control patients versus CD patients (left) and control patients versus UC patients (right). (Image: Costello et al. [2])

In a study published in *PLoS Medicine*, Schreiber and coworkers have used a comprehensive gene expression approach to find new genes and pathways relevant to the pathophysiology of IBD [2]. They took a close look at the genes differentially expressed in mucosal biopsies taken from the sigmoidal colons of ten individuals with UC, ten individuals with CD, and 11 control individuals (Figure 1). The genes were studied by hybridizing RNA from these biopsies to membranes spotted with some 23,000 unique transcripts from the human genome. One of the strengths of this study is the access to tissue involved in the disease process, although the biopsies represent a mixed population of cells.

The authors initially examined active disease tissues (i.e., from untreated patients), which comes with the risk of mainly seeing the consequence of the disease rather than the cause. Their experimental design is extremely robust for obtaining small but significant differences in gene expression: ten measurements per gene were made for each sample, as the genes were spotted in duplicate and the experiments were repeated five times. The authors were able to identify genes with at least a 1.2-fold change between groups, differences much smaller than commonly reported for these types of studies. Hence, the number of genes differentially expressed was rather large: 378 genes unique to CD, 150 genes unique to UC, and 122 genes differentially expressed in both groups, compared to normal control tissue. Although both UC and CD share a general inflammation profile, these results strengthen earlier suggestions that CD and UC are, at the molecular level, two related yet different forms of chronic intestinal inflammation. Follow-up studies indicated that most of these differentially expressed genes may not be IBD-specific, but rather a consequence of colon inflammation.

Three other microarray studies on IBD have been published, but there is little overlap between the individual genes identified [3–5]. Although the experimental designs of these four studies differ significantly, it is interesting that the studies all point to the involvement of similar biological processes: immune-related processes, oncogenesis/cell proliferation/growth, and structure/permeability-related processes.

## What Can Be Learned from These Studies?

Gene-expression profiling studies appear to hold much promise. In cancer studies, this promise has been met with the identification of many profiles that can be used to classify different tumor stages or to predict response to therapy. The gene-expression changes in tumor tissues are, in general, much more pronounced than those seen in other diseased tissues. A number of potentially interesting, new leads for therapeutic targets or disease diagnosis have come from Costello et al.'s study. Upregulation of cancer-related genes, such as *TFF1* and the gene that encodes the Wnt signaling molecule CSNK1D, is very specific to the UC profile, and may point toward these genes playing a role in the increased risk of malignant transformation for patients with a long history of UC. Uthoff et al. also suggested a potential role for the Wnt signaling pathway in UC carcinogenesis in an earlier but much smaller study [6], but this needs to be further investigated. It is encouraging to learn that many of the genes now being identified confirm the ideas on disease pathogenesis that have developed recently as a result of genetic studies, namely, that there is impaired integrity of the epithelial barrier. Hence, Costello et al.'s study may further our understanding of the underlying disease mechanism.

Another interesting avenue for gene expression data is its use in selecting novel disease gene candidates. Costello et al. have identified 59 differentially expressed genes that map to IBD linkage regions. Genetic variants contributing to a complex disease like IBD are expected to be regulatory variants resulting in differential gene expression, rather than structural variants (amino acid substitutions). Although the majority of these 59 genes are more the consequence of the disease than the cause, current technology and recent accessibility to large numbers of single nucleotide polymorphisms make it feasible to test them for genetic association.

## Looking Toward the Future

As gene expression studies mainly generate hypotheses, they provide a basis for further detailed gene function studies. However, the majority of genes included in these types of studies have an unknown function or a limited functional annotation at best, making it difficult to identify functional relationships between genes. This situation is expected to change with the ongoing large-scale functional genomics studies now being conducted. Nevertheless, genes with an unknown function can still be excellent candidate genes for testing by genetic association. Since expression studies mainly highlight the perturbed pathways involved, and genetic studies point to critical players in disease pathways, it is obvious that much can be gained from integrating the two. As Schreiber's research group was instrumental in identifying *DLG5* as one of the IBD genes, this group is in an excellent position to perform both types of studies [7].

A future application might be to use disease-specific signatures as predictive diagnostic tools for distinguishing CD from UC. Since mucosal biopsies are invasive and burdensome for the patients, it would be interesting to determine whether similar patterns of expression can also be obtained from peripheral blood.

## Abbreviations

CD: Crohn disease
IBD: inflammatory bowel disease
UC: ulcerative colitis
